# Evaluation of vital genes correlated with CD8 + T cell infiltration as prognostic biomarkers in stomach adenocarcinoma

**DOI:** 10.1186/s12876-023-03003-y

**Published:** 2023-11-17

**Authors:** Dun Pan, Hui Chen, Jiaxiang Xu, Xin Lin, Liangqing Li

**Affiliations:** Department of Gastrointestinal Surgery, The First Affiliated Hospital of Fujian Medical University, No.20, ChaZhong Road, TaiJiang District, Fuzhou, 350000 Fujian Province China

**Keywords:** Stomach adenocarcinoma, CD8 + T cells, Immune infiltration, Tumor microenvironment, Prognostic biomarkers

## Abstract

**Background:**

Infiltration of CD8 + T cells in the tumor microenvironment is correlated with better prognosis in various malignancies. Our study aimed to investigate vital genes correlated with CD8 + T cell infiltration in stomach adenocarcinoma (STAD) and develop a new prognostic model.

**Methods:**

Using the STAD dataset, differentially expressed genes (DEGs) were analyzed, and co-expression networks were constructed. Combined with the CIBERSORT algorithm, the most relevant module of WGCNA with CD8 + T cell infiltration was selected for subsequent analysis. The vital genes were screened out by univariate regression analysis to establish the risk score model. The expression of the viral genes was verified by lasso regression analysis and in vitro experiments.

**Results:**

Four CD8 + T cell infiltration-related genes (CIDEC, EPS8L3, MUC13, and PLEKHS1) were correlated with the prognosis of STAD. Based on these genes, a risk score model was established. We found that the risk score could well predict the prognosis of STAD, and the risk score was positively correlated with CD8 + T cell infiltration. The validation results of the gene expression were consistent with TCGA. Furthermore, the risk score was significantly higher in tumor tissues. The high-risk group had poorer overall survival (OS) in each subgroup.

**Conclusions:**

Our study constructed a new risk score model for STAD prognosis, which may provide a new perspective to explore the tumor immune microenvironment mechanism in STAD.

**Supplementary Information:**

The online version contains supplementary material available at 10.1186/s12876-023-03003-y.

## Introduction

 Gastric cancer (GC) is the third leading cause of cancer death worldwide [1–3]. According to latest cancer statistics information, 1,958,310 new cancer cases and 609,820 cancer deaths are projected to occur in the United States [4]. The vast majority (about 90%) of GCs are adenocarcinomas arising from the glands in the most superficial or mucosal layers of the stomach [5]. The early symptoms of gastric adenocarcinoma (STAD) are not obvious, but the late symptoms are poor digestion, anorexia nervosa and celialgia [6]. Despite major advances in the treatment of STAD, the prognosis of STAD patients remains unsatisfactory [7–9]. In recent years, the development of immunotherapy has a significant impact on the treatment of STAD [10]. It is worth noting that the immunotherapy effect mainly depends on the immune response [11], which is markedly affected by the tumor microenvironment [12]. CD8 + T cells are core effector cells in the tumor microenvironment, and highly infiltrating CD8 + T cells have prognostic value in most tumors [13]. However, the mechanism of CD8 + T cell infiltration in the STAD tumor microenvironment remains unclear. Therefore, identification of novel biomarkers correlated with CD8 + T cell infiltration is helpful to explore the mechanisms of immune infiltration in STAD.

There are various deconvolution methods for quantifying the cellular composition of immune cells, such as xCell, TIMER, EPIC, QuanTIseq and CIBERSORT. xCell, a novel gene signature-based method, can be used to infer 64 immune and stromal cell types [14]. TIMER was able to estimate the abundance of 6 immune cell types in 32 cancer types [15]. EPIC is to estimate the proportion of immune and cancer cells from bulk tumor gene expression data, and can accurately detect all major cell types in tumors [16]. QuanTIseq is a method to quantify the fractions of ten immune cell types from bulk RNA-sequencing data [17]. CIBERSORT is a common method for calculating immune cell infiltration, providing expression data for 22 common immune infiltrating cells [18]. CIBERSORT consistently outperformed other methods in some cases, and unknown content and lab-specific factors had little effect on CIBERSORT performance [19]. It was successfully applied to estimate the level of immune cell infiltration in various cancers, including renal cell carcinoma [20], colon cancer [21]. Therefore, CIBERSORT was selected in our study to quantify the cellular composition of immune cells.

In our current research, we obtained differentially expressed genes (DEGs) in the Cancer Genome Atlas (TCGA) database. The important modules and genes associated with CD8 + T cell infiltration were identified with WGCNA and CIBERSORT methods. The risk score model was constructed by univariate regression analysis and LASSO regression analysis. And the expression levels of these genes in risk score model were verified in validation set and in vitro experiment. This may provide a theoretical basis for the exploration of prognostic biomarkers of STAD.

## Materials and methods

### Data set sources and preprocessing

All data in this study were obtained from the TCGA and GEO database. In TCGA database, STAD expression and clinical data from 375 STAD patients and 32 adjacent normal tissue samples were downloaded from UCSC Xena. After excluding samples with overall survival (OS) less than 30 days, 335 STAD samples were ultimately included in the TCGA cohort. TCGA dataset was used to screen vital genes and the construct the prognostic model. The GSE26899 dataset and GSE29272 dataset were downloaded from the GEO database for the expression validation of vital genes. The GSE26899 dataset included 96 tumor tissues and 12 adjacent normal tissue samples, and the GSE29272 dataset included 134 tumor tissues and 134 adjacent normal tissue samples. GSE84437 dataset was downloaded from the GEO database to verify the prognostic ability of the constructed model, which included 431 tumor samples. Among them, the TCGA dataset was regarded as the training set, and the GEO dataset was regarded as the validation set.

### Identification of DEGs

DEGs were identified using the limma package in R, and visualized with volcano plots and heatmaps. False discovery rate (FDR) < 0.05 and |log_2_ Fold Change (FC)| >2 were considered as the criteria for identifying DEGs. Heatmaps and volcano plots were drawn by the R packages “pheatmap” and “ggplot”, respectively.

### WGCNA

WGCNA is a typical phylogenetic algorithm to describe the correlation patterns between gene expression profiles and build gene co-expression networks. The DEGs co-expression network analysis was performed by the R package “WGCNA”, and a scale-free gene co-expression network was built. First, outliers were detected by clustering sample data with the “hclust” function. Then, the “pickSoftThreshold” function was applied to select a suitable soft-threshold power regulator to construct a scale-free topology with a soft threshold of 5. The adjacency matrix was calculated from this value, which was transformed into a topological overlap matrix (TOM) and the corresponding dissimilarity matrix (1-TOM). Genes were clustered by the mean linkage hierarchical clustering method. The minimum number of genes per gene network module was set to 30 based on the criteria of the hybrid dynamic clipping tree method. The signature genes of each module were calculated in turn, and the modules were clustered.

### Analysis of immune infiltration and functional enrichment

The proportion of immune cells in the sample was calculated by “CIBERSORT”. Correlations between WGCNA module genes and T cell subtypes were calculated using Pearson’s test. The modules most significantly associated with CD8 + T cells were used for subsequent analyses. Enrichment analysis of genes in the modules most significantly associated with CD8 + T cells was performed by the R package “clusterProfiler” with a threshold of *p* < 0.05.

### Construction and verification of prognostic model

The genes significantly associated with CD8 + T cells in the module were analyzed by univariate Cox regression, and the prognostic genes were screened (*p* < 0.05). The prognostic model was further constructed by LASSO Cox regression analysis. The risk score was calculated as follows: risk score = (ßA × gene A expression) + (ßB × gene B expression) + (ßN × gene N expression). The genes used to construct the risk score were defined as vital genes. The median risk score served as a cut-off point to divide patients into high- and low-risk groups. The risk score was also calculated in the GSE84437 validation set. OS curves of risk score were analyzed by Kaplan-Meier, and the accuracy of the model was verified using the time-dependent ROC curve. Additionally, univariate Cox regression analysis and multivariate Cox regression analysis were used to identify whether the risk score was an independent prognostic factor for OS in patients with STAD.

### Expression and verification of vital genes

The expression of genes in prognostic model was analyzed in the TCGA validation set by rank sum test. It was displayed by boxplot. Furthermore, we also validated the expression of genes in the GSE26899 and GSE29272 validation sets. The Human Protein Atlas (HPA) (https://www.proteinatlas.org/) provided immunohistochemical (IHC) results of genes in adjacent normal tissues and tumor tissues.

### In vitro expression validation of vital genes by real time polymerase chain reaction (RT-PCR)

Nine STAD patients were recruited from First Affiliated Hospital of Fujian Medical University. Tumor tissues and adjacent normal tissues were collected from 9 STAD patients. Clinical information of individuals was displayed in Table 1, mainly including the age, gender, stage, grade, drinking, smoking and family history.

**Table 1:** Clinical Information

Number	Age (years)	Gender	Height (cm)	Weight (Kg)	BMI	Clinical stages			Stage	Grade	Tumor metastasis	Alcohol history	Smoking history	Family history
						T	N	M						
1	54	Female	158	61	24.44	T4a	N2	M0	IIIB	G3	No	No	No	No
2	72	Male	164	50	18.59	pT4a	N0	M0	?B	G3	No	Yes	Yes	No
3	68	Male	165	56	20.57	T4a	N3	M0	IIIC	G3	No	No	No	No
4	68	Male	167	60	21.51	T2	N0	M0	IB	G2	No	No	No	No
5	72	Female	150	47.5	21.11	T4a	N0	M0	?B	G2	No	No	No	No
6	68	Male	162	58	22.10	T1a	N0	M0	IA	G1	No	No	No	No
7	68	Male	170	70	24.22	T3	N0	M0	?A	G3	No	No	No	No
8	61	Female	155	42	17.48	T1a	N0	M0	IA	G4	No	No	No	No
9	59	Male	168	60	21.25	T3	N2	M0	IIIA	G3	No	No	No	No

Inclusion criteria of STAD patients: (1) The patients were initially diagnosed with STAD; (2) The patients did not undergo other therapy before diagnosis; (3) The patients had no other malignant tumor; (4) The patients had no other autoimmune diseases; (5) The patients were 18 to 70 years old. The exclusion criteria of STAD patients: (1) Patients had other malignancy; (2) Patients received other treatment before surgery; (3) Patients had incomplete clinical data; (4) Patients had a history of STAD; (5) Patients with recurrence. The Ethics Committee of First Affiliated Hospital of Fujian Medical University approved this study (2,020,219). Informed consent of patients and their families was obtained.

Total RNA was extracted from tissue samples using TRIzol® Reagent. FastKing cDNA first-strand synthesis kit (KR116) was used for reverse transcription of mRNA, and Gene-9660 fluorescence quantitative PCR instrument was used for relative quantitative analysis of data by 2^-??ct^ method. GAPDH and ACTB was used for internal reference genes. The primer information for RT-PCR is shown in Table 2.

**Table 2:** Primer sequence in the RT-PCR

Primer name	Primer sequence (5’ to 3’)
GAPDH-F (Internal reference)	GGAGCGAGATCCCTCCAAAAT
GAPDH-R (Internal reference)	GGCTGTTGTCATACTTCTCATGG
ACTB-F (Internal reference)	CATGTACGTTGCTATCCAGGC
ACTB-R (Internal reference)	CTCCTTAATGTCACGCACGAT
CIDEC-F	TTCCCCAGTGAAGGACTGACT
CIDEC-R	GACCAGTCTGGATGGGCTAAG
EPS8L3-F	CAGAAGCTGTTCGAGATGGATG
EPS8L3-R	GCTGTCTAGGCGGTAAGAGTC
MUC13-F	AGCGCTTGTCAGAGAGGTG
MUC13-R	ACCTCCACAGTTGATGCGT
PLEKHS1-F	GACGTGGTGGTTCATGCCT
PLEKHS1-R	CATGTGCCACAATGCCCAG

### Subgroup analyses to evaluate model performance

Risk score for different subgroups of tumor (T), node (N), metastasis (M), and grade (G) was assessed in the TCGA and GEO datasets to test the performance of the model. In addition, the Kaplan-Meier method and the log-rank test were applied to assess the ability of prognosis prediction in different subgroups. *p* < 0.05 was statistically significant.

### Evaluation of drug therapy and risk score

To assess the response of risk score for drug therapy, the “pRRophetic” R package was applied to calculate the half-maximal inhibitory concentration (IC50) of samples. Based on clinical recommendations, molecular drugs such as Axitinib, Dasatinib, Etoposide, Midostaurin, Pyrimethamine and Sunitinib were selected as drug candidates. IC50 was compared between high- and low-risk groups by Wilcoxon signed-rank test.

### Statistical analysis

All statistics were performed with R software. “Limma” and “WGCNA” were applied to screen genes correlated with CD8 + T cells. Univariate Cox regression analysis was performed by the “survival” and “survminer” package. Lasso analysis and model construction were performed by the “glmnet” software package. The Wilcox test was applied to determine statistical differences. Kaplan-Meier curves were plotted and log-rank was applied to test for significant differences in OS among groups. ROC analysis was applied to assess the prognostic performance of risk score. ROC-AUC was an indicator for judging the accuracy of prognosis. *P* < 0.05 was statistically significant in all analyses.

## Results

### Identification of DEGs and WGCNA

**Fig. 1:**
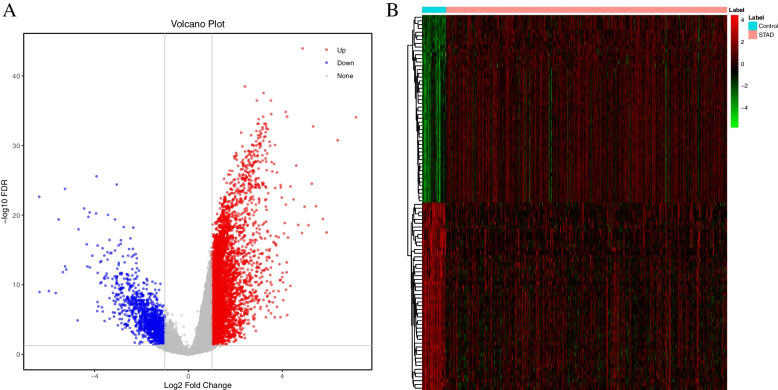
Volcano plot (**A**) and heat map of top 100 DEGs (**B**)

**Fig. 2:**
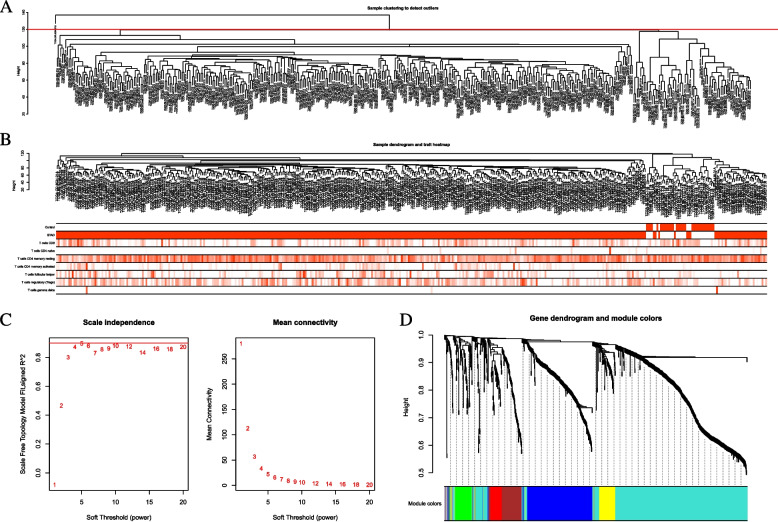
Weighted Gene Co-expression Network Analysis (WGCNA) analysis.  **A** Sample clustering to detect outliers; **B** Sample dendrogram and scoring feature heatmap; **C** Soft threshold screening: **D** Genes clustering

In total, 1013 DEGs were identified, including 700 up-regulated genes and 313 down-regulated genes (Fig. 1A). In addition, the heatmap of the top 100 DEGs is shown in the Fig. 1B. The 1013 DEGs were subjected to construct a weighted gene co-expression network. First, the samples were clustered by the mean linking method in WGCNA (Fig. 2A). 120 was chosen as the cutting tree height to remove outliers (red line). After re-clustering, the number of samples below the red line was 406. The dendrogram and rating feature heatmap for the 406 samples in the study are shown in Fig. 2B. ß = 5 was chosen as the soft threshold to construct the scale-free network (Fig. 2C), and 7 modules were confirmed (Fig. 2D).

### Identification of key modules

**Fig. 3:**
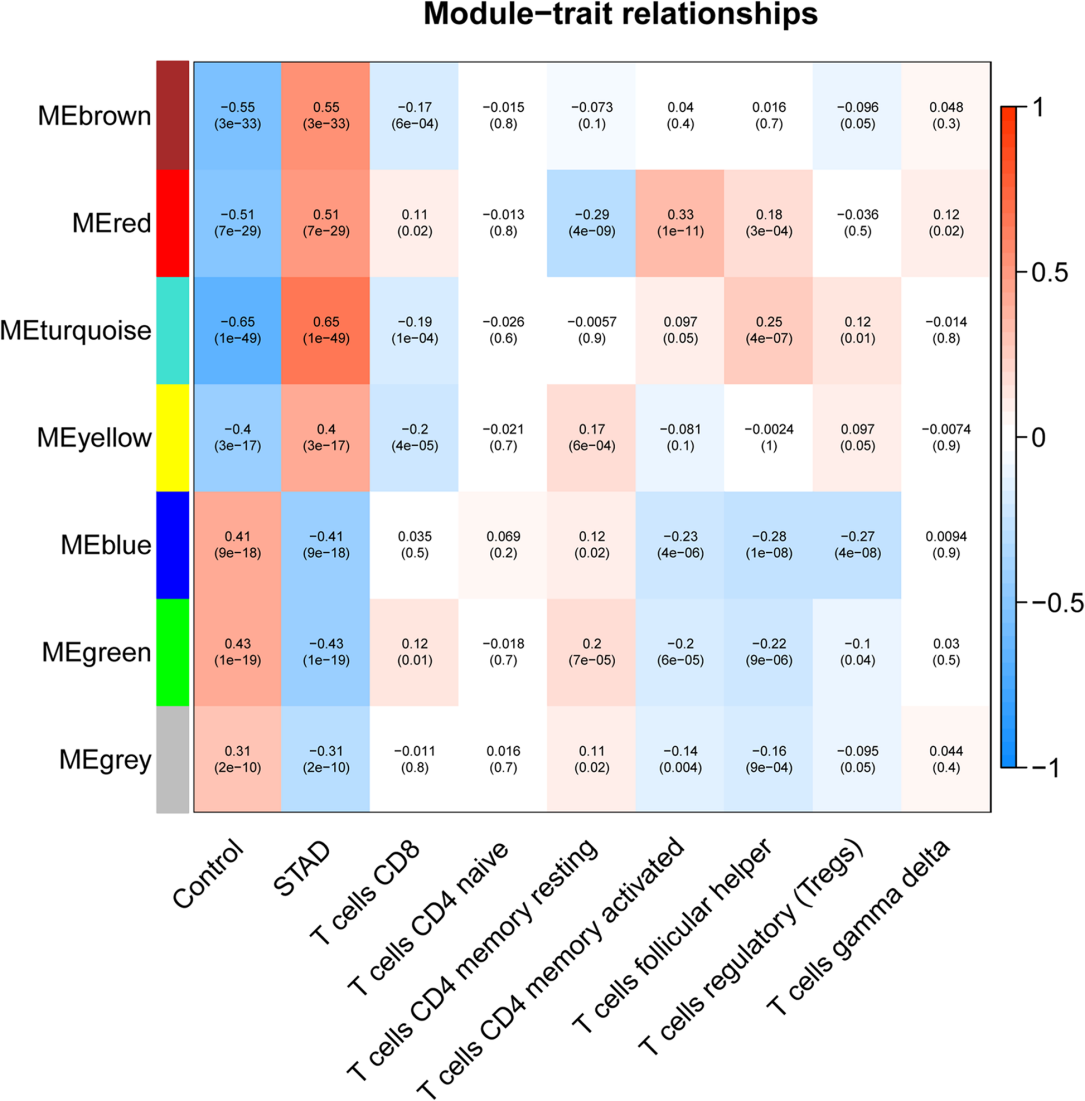
Correlation of T cell subtypes infiltration with different gene modules

The proportions of 7 T cell subtypes including CD8 + T cells in the samples were calculated by CIBERSORT and their association with the WGCNA module were analyzed. The highest correlation was identified between genes in the yellow module (65 genes) and CD8 + T cells. Therefore, genes in yellow module were selected for subsequent analysis (Fig. 3).

### Functional enrichment analysis of genes

**Fig. 4:**
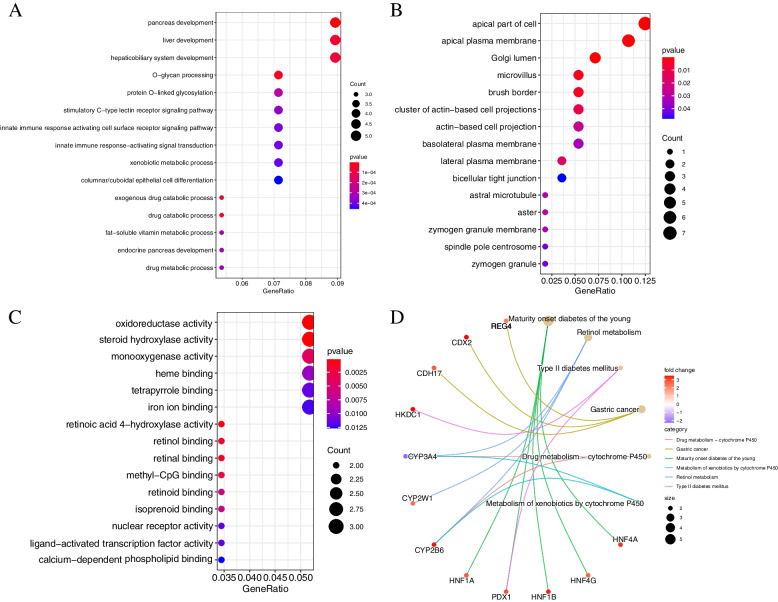
GO and KEGG enrichment analysis of genes in yellow module. **A** Biological process (BP); **B** Cellular component (CC); **C** Molecular function (MF); **D** KEGG enrichment analysis (https://www.kegg.jp/kegg/kegg1.html). Appropriate copyright permission’ to use the signalling pathways was obtained

Gene Ontology (GO) and the Kyoto Encyclopedia of Genes and Genomes (KEGG) function enrichment analysis were performed on the 65 genes in the yellow module using the R package “clusterProfiler” at a screening criteria of *p* < 0.05. Top 15 of biological process (BP), cellular component (CC) and molecular function (MF) analyzed by GO enrichment are shown in Fig. 4A-C, respectively. DEGs were mostly enriched in the BP of O-glycan processing and protein O-linked glycosylation, and CC of apical part of cell. KEGG analysis indicated that DEGs were mostly enriched in maturity onset diabetes of the young, retinol metabolism and GC. The top 5 enriched pathways were used to construct a network. From the network, regenerating family member 4 (REG4), caudal type homeobox 2 (CDX2), cadherin 17 (CDH17) were supposed to have a direct relationship with GC (Fig. 4D).

### Construction and verification of a prognostic model

**Fig. 5:**
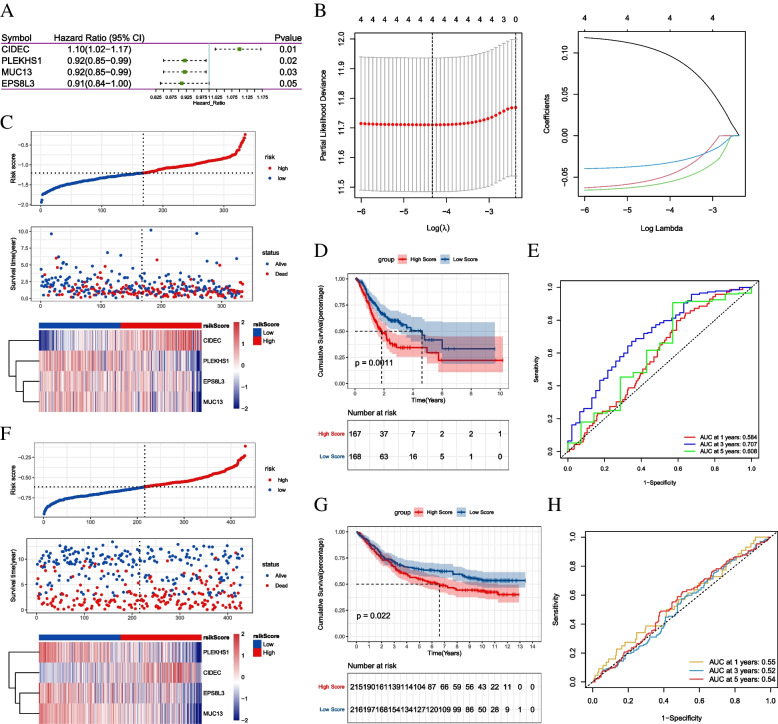
Construction and validation of a prognostic model of CD8 + T cell infiltration-related genes.  **A** Screening of vital genes; **B** LASSO Cox regression analysis; **C** Distribution map of risk score in TCGA training set; **D** Survival Curve in TCGA training set; **E** ROC curve in TCGA training set; **F** Distribution map of risk score in GSE84437 validation set; **G** Survival curve in GSE84437 validation set; **H** ROC curve in GSE84437 validation set

After univariate Cox regression analysis of 65 genes, 4 prognostic genes were obtained (*p* < 0.05) (Fig. 5A). Afterwards, a four-gene risk model consisting of CIDEC, EPS8L3, MUC13 and PLEKHS1 was constructed (Fig. 5B). Risk score was calculated for each tumor sample using the coefficients obtained by the LASSO algorithm. The formula was as follows: risk score = CIDEC * 0.104 + PLEKHS1* (-0.035) + MUC13 * (-0.059) + EPS8L3 * (-0.052). STAD patients were divided into high- and low-risk groups based on a median risk score. Patients in the low-risk group had a higher survival proportion (Fig. 5C). Kaplan-Meier survival analysis showed significantly lower OS in the low-risk group patients (Fig. 5D). To evaluate the predictive efficiency of the model in 1-, 3-, and 5-year survival, ROC curves were performed on the training set, indicating that the prognostic model has good sensitivity and specificity (Fig. 5E).

To further assess the robustness of the prognostic model constructed by the four vital genes, the risk score was calculated in the GSE84437 validation set. Patients with low-risk score had longer OS, which was in line with the results in training set (Fig. 5F). Kaplan-Meier survival analysis showed that the OS of patients in the low-risk group was lower than that in the high-risk group (Fig. 5G). Survival prediction by risk score was assessed by ROC analysis at 1, 3 and 5 years (Fig. 5H).

### Expression and verification of vital genes

**Fig. 6:**
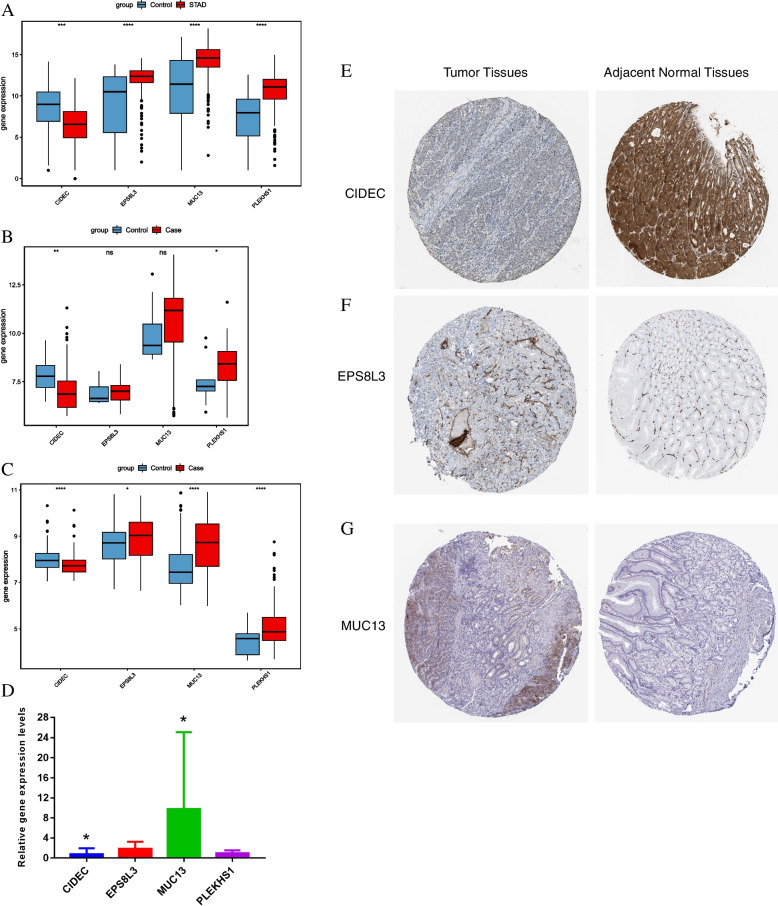
Expression of CD8 + T cell infiltration-related genes in tumor tissues and tumor adjacent tissues.  **A** Expression of genes in TCGA training set; **B** Expression of genes in GSE26899 validation set; **C** Expression of genes in GSE29272 validation set; **D** Expression of genes in RT-PCR; **E**-**G** Immunohistochemical analysis of genes.  *p < 0.05; **p < 0.01; ***p < 0.001 and ****p < 0.0001; ns, not significant

In addition, the relative expression of four vital genes between tumor tissues and adjacent normal tissues was explored by the TCGA dataset and further validated by the GSE26899 and GSE29272 dataset. CIDEC was significantly down-regulated in tumor tissues, while EPS8L3, MUC13 and PLEKHS1 were significantly highly expressed in tumor tissues (Fig. 6A-C). In addition, RT-PCR results displayed that CIDEC was significantly reduced in tumor tissues, MUC13 was significantly increased in tumor tissues, EPS8L3 showed an up-regulated tendency, while PLEKHS1 was down-regulated in tumor tissues (Fig. 6D). We speculated that the small sample size may cause this difference. IHC further confirmed the differential expression of CIDEC, EPS8L3 and MUC13 in tumor tissues and adjacent normal tissue. The expression of CIDEC in tumor tissue was significantly lower than that in adjacent normal tissues (Fig. 6E), while the expression of EPS8L3 and MUC13 was significantly higher in tumor tissues (Fig. 6F-G).

### Risk score was an Independent prognostic factor for STAD

**Fig. 7:**
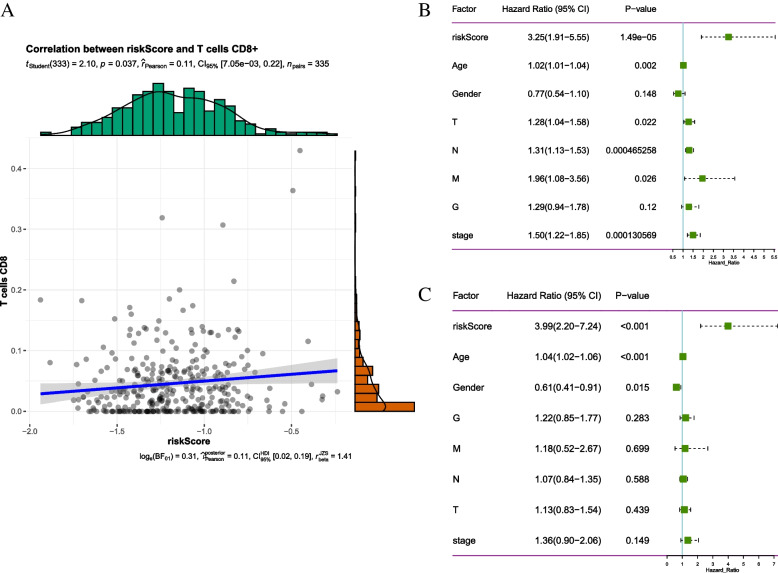
Correlation of risk score with CD8 + T cells and OS.  **A** Correlation analysis; **B** Univariate regression analysis; **C** Multivariate regression analysis

The correlation analysis shows that the risk score was positively connected with CD8 + T cell infiltration (Fig. 7A). Univariate analysis revealed that, except for gender and G stage, the other factors were correlated with OS (Fig. 7B). Incorporating these factors into multivariate analysis, risk score remained significantly related to OS (Fig. 7C). These results indicate that risk score was an independent prognostic factor for STAD.

### Correlation analysis of vital genes and immune cell subtypes

**Fig. 8:**
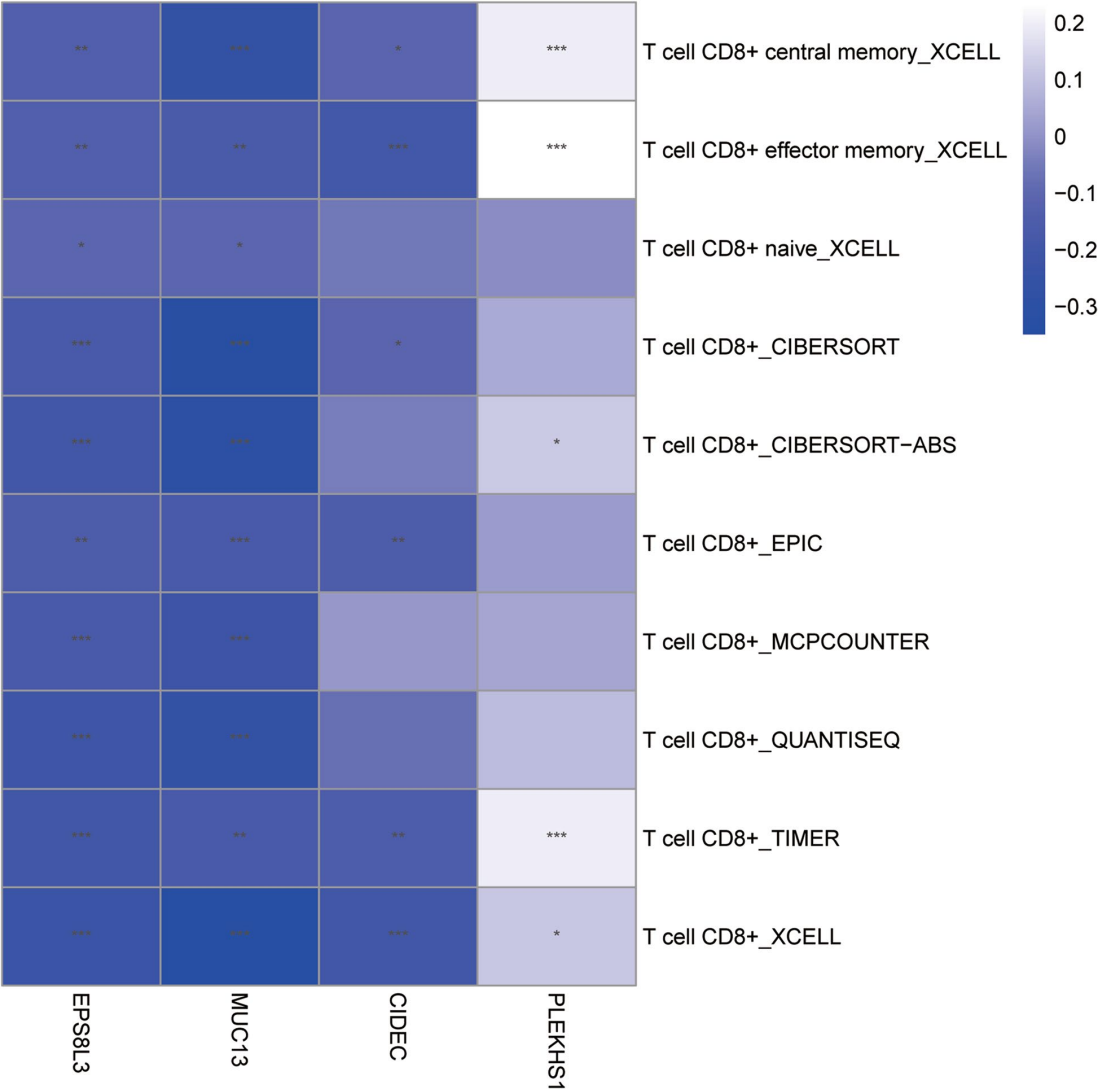
Correlation analysis of genes and immune cell subtypes in the TIMER database

The correlation analysis of vital genes and CD8 + T cells was performed based on the TIMER2.0 database. With XCELL algorithm, TIMER algorithm, CIBERSORT algorithm and EPIC algorithm, CIDEC, EPS8L3, and MLC13 were remarkable negatively correlation with CD8 + T cells, but PLEKHS1 were positively correlation with CD8 + T cells (Fig. 8).

### Subgroup analysis

**Fig. 9:**
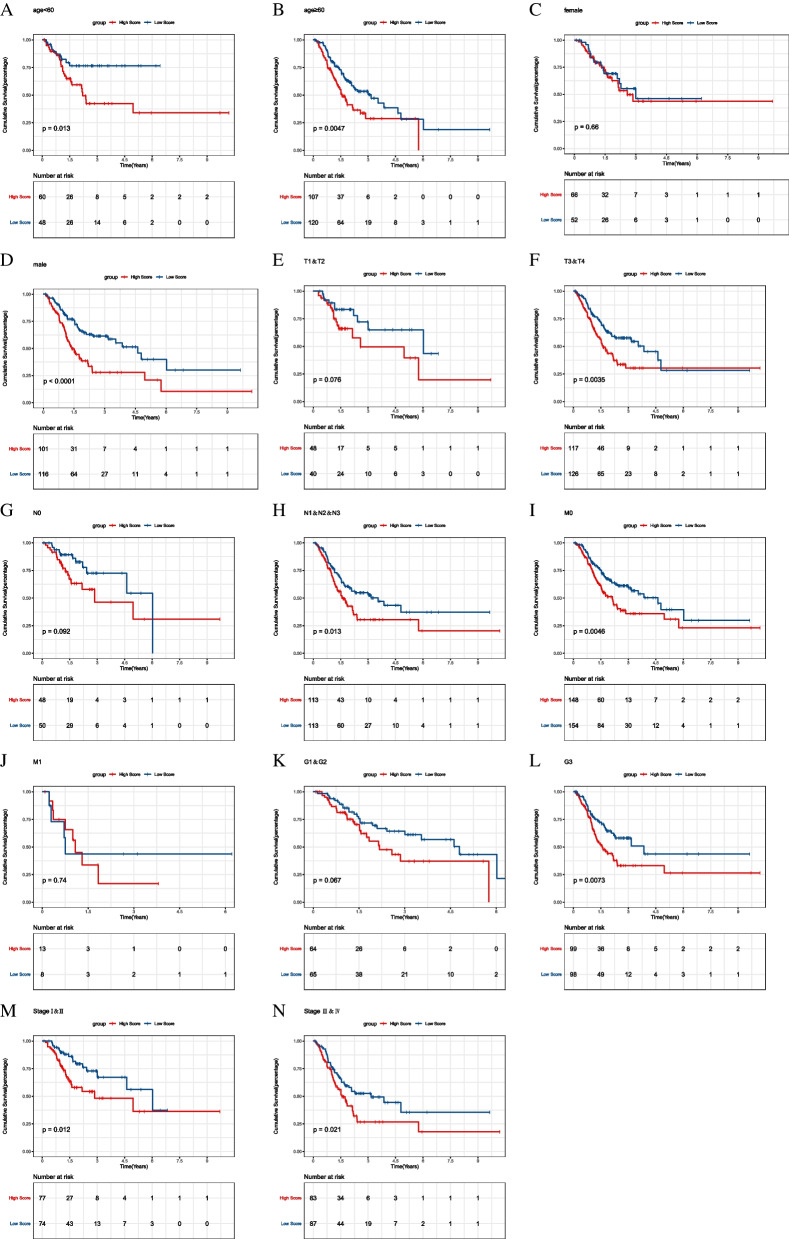
Subgroup survival analysis in TCGA training set

**Fig. 10:**
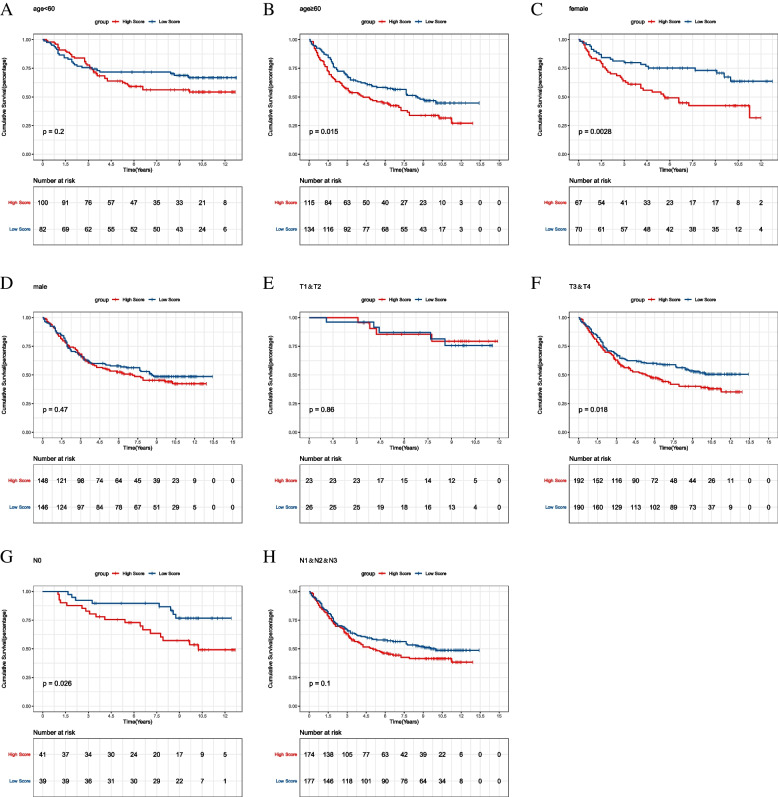
Subgroup survival analysis in GSE84437 validation set

Kaplan-Meier curves were plotted for subgroups including age, gender, clinical stages in the training set. In the clinical indicators such as age = 60, T3 + T4, the high-risk groups have poor survival prognosis (Fig. 9). Survival was significantly different in age = 60 and T3 + T4 subgroups between high- and low-risk groups in GSE84437 validation set (Fig. 10). The result suggests that risk score model could identify outcomes in different subgroups of patients.

###  Evaluation of drug therapy and risk score

**Fig. 11:**
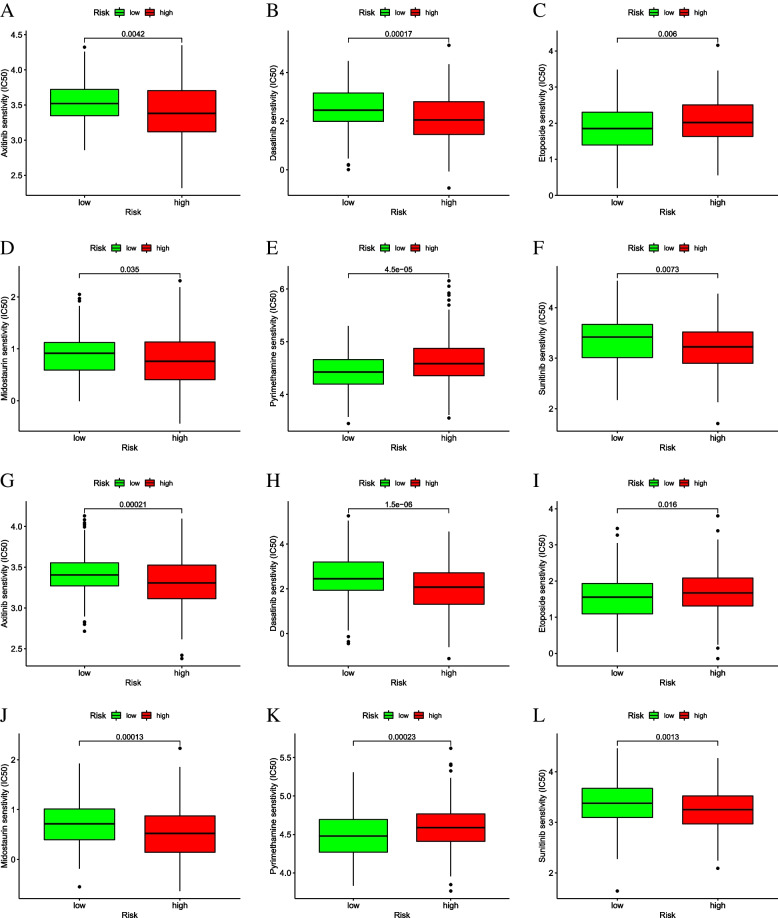
Sensitivity to drugs in high- and low-risk groups of risk score.  **A**-**F**: in TCGA training set; **G**-**I**: in GSE84437 validation set

The IC50 of Axitinib, Dasatinib, Midostaurin, and Sunitinib was higher in the low-risk group and the IC50 of Etoposide and Pyrimethamine was lower in low-rish group based on the evaluation of risk score model and drug treatment sensitivity (Fig. 11). In addition, the IC50 of Cisplatin and Docetaxel, which are commonly used in the treatment of gastric adenocarcinoma, was higher in the low-rish group in GEO dataset (Supplementary Fig. 1). These results suggest that risk score has potential predictive value for chemotherapy and targeted therapies.

## Discussion

STAD is a heterogeneous malignancy with high risk of locoregional recurrence and distant metastasis after surgery [22, 23]. In recent years, increasing studies have explored the prognostic biomarkers of STAD. For instance, Jiang et al. mined the differentially expressed genes (DEGs) in the early stage of STAD and constructed a prognostic signature with 10 early-stage specific mRNAs [24]. Shen et al. constructed a STAD prognosis prediction model based on 8 chemotherapy-related characteristic genes to provide a reference for the treatment and prognosis improvement of STAD patients [25]. Immunotherapy is increasingly recognized for its potential therapeutic effects on various tumors [26, 27]. CD8 + T cells are the central effector cells of anti-tumor immunity [28]. Identification of vital genes associated with CD8 + T cell infiltration may provide novel ideas for the prevention and treatment of STAD.

CD8 T cell infiltration is correlated with better clinical outcomes in many cancers. Triple Negative Breast Cancer (TNBC) patients with high infiltration of CD8 T cells had better survival and highly immunoreactivity [29]. High expression of CD8 + T lymphocytes has a strong association with prognostic in GC [30]. The study by Huang et al. in breast cancer further confirms that CD8 T cells are regarded as the main effector cells of anti-tumor immunity [31]. Therefore, tumor-infiltrating CD8 T cells may be an important biomarker for predicting cancers.

In total, 700 up- and 313 down-regulated DEGs were identified in STAD. Subsequently, essential modules and genes most significantly associated with CD8 + T cells were confirmed. Finally, we identified four vital genes (CIDEC, EPS8L3, MUC13 and PLEKHS1) correlated with prognosis and CD8 + T cell infiltration by Lasso Cox regression analysis.

Cell death-inducing DFFA-like effctor c (CIDEC) is predominantly expressed in white adipose tissue, which is essential in lipid metabolism and energy regulation [32]. Relevant literature studies have revealed that CIDEC is over-expressed in various cancers, such as hepatocellular carcinoma [33] and clear cell renal cell carcinoma [34]. Wang et al. reported that the expression of CIDEC increase may reduce fatty acid oxidation and promotes de novo lipogenesis in human adenovirus-infected primary cultured skeletal muscle [35]. Nevertheless, there is no study about the expression of CIDEC in STAD. The low expression of CIDEC in STAD was first found in this study. Epidermal growth factor receptor kinase substrate 8 like 3 (EPS8L3) has reported in various cancers, sunch as live cancer, pancreatic cancer and gastric cancer, which involved in regulating cell proliferation, differentiation and migration [36–38].The expression of EPS8L3 is markedly up-regulated in liver cancer tissues and cell lines, and patients with high expression of EPS8L3 have shorter survival [36, 39]. In the hepatocellular carcinoma (HCC) cell line, overexpression EPS8L3 can enhance cell proliferation [40]. This indicates that EPS8L3 is correlated with poor clinical prognosis. Mucins are recognized as potential oncogenes and possible therapeutic targets in various malignancies [41, 42]. MUC13, a high molecular weight transmembrane glycoprotein, is frequently overexpressed in various epithelial cancers [43]. Shimamura et al. detected up-regulation of MUC13 at GC’s mRNA and protein levels [44]. Cai et al. showed that the expression of MUC13 increase can enhance GC cell proliferation and invasion [45]. In addition, MUC13 can be used as a marker for early cancer screening, providing a promising target for targeted therapy. PLEKHS1 is up-regulated in the majority of cancers. Highly recurrent mutations in PLEKHS1 may lead to tumorigenesis [46]. Chessa et al. reported that PLEKHS1 can escape homeostasis, actovate the PIP3 signaling, and support tumour progression in cells absenting PTEN [47]. Furthermore, Xing et al. showed that overexpression PLEKHS1 enhanced anaplastic thyroid carcinomas (ATC) invasion in cell experiment [48]. Besides, Over-expressed PLEKHS1 increases the risk of disease progression in bladder cancer [49]. PLEKHS1 is one of the up-regulated DEGs in hepatocellular carcinoma and a poor prognostic indicator [50]. Therefore, the expression of PLEKHS1 may be correlated with cancer prognosis.

We built a risk score model based on four genes. Risk score was an independent prognostic factor for OS in patients with STAD and was positively connected with CD8 + T cell infiltration. These four genes may play a role in CD8 + T cell infiltration in the immune microenvironment. CD8 + tissue-resident memory T (Trm) cells depended on fatty acid oxidation for cell survival in STAD patients. Absention of fatty acid leads to Trm cell death [51]. Furthermore, Joseph et al. evidenced that CD8 + T cells suppress tumour metastasis in mouse tumour models [52]. Correlation analysis indicated that CIDEC, EPS8L3, and MLC13 were remarkably negatively correlated with CD8 + T cells. Li et al.‘s study showed a correlation between CD8 T cells and CIDEC [53]. The correlation between the other two genes and CD8 T cells in tumours has not been reported. Based on the above results, we speculated that CIDEC expression may influence fatty acid metabolism and reduce the number of CD8 T cells. In addition, EPS8L3, MLC13, and PLEKHS1 can enhance tumour invasion. Therefore, CIDEC, EPS8L3, MLC13, and PLEKHS1 may reduce the number of CD8 T cells and increase the aggressiveness of STAD, leading to poor prognosis. CIDEC, EPS8L3, MLC13 and PLEKHS1 may be potential prognostic factors for STAD and can be used to assess the level of immune cell infiltration in tumour tissues. However, we look forward to collecting more samples for mechanistic analysis to demonstrate the correlation between these genes and CD8 + T cell infiltrating tumours in future studies.

Additionally, we assessed the sensitivity of the risk score model to drug therapy. IC50 is the concentration at which an anticancer drug kills half of the inhibitory concentration of cancer cells [54]. It helps to quantify the therapeutic ability of a drug to induce apoptosis in cancer cells, which is inversely proportional to the sensitivity of small molecule drugs [55]. Among them, the IC50 of Axitinib, Dasatinib, Midostaurin and Sunitinib in the low-risk group was significantly higher than that in the high-risk group. In contrast, the IC50 of Etoposide and Pyrimethamine was higher in low-risk group. Above results suggested that the drugs may have a good therapeutic effect in STAD patients. The results of related studies also showed that these drugs had lower IC50 values in STAD high-risk group compared with low-risk group, suggesting that these drugs may be more sensitive to high-risk patients [55–58]. In addition, the IC50 of Cisplatin and Docetaxel in GEO dataset was lower in higer-rick group, suggesting that Cisplatin and Docetaxel are certain effective in high-risk subgroups of gastric adenocarcinoma patients. In summary, we speculate that the four-gene risk score model can not only divide patients into different risk groups, but also may help clinicians in clinical decision making for patients in different risk groups.

However, some limitations in our study should be pointed out. First, the identification of four risk genes and construction of the risk score model were based on the systematic bioinformatics analysis of gene expression profiles and public database. Further experiments, such as immunostaining, are needed to evaluate the association between risk score and the extent of CD8 + T cells. Second, the mechanisms underlying the impact of these genes on CD8 + T cell infiltration were not investigated in this study; which will be included in our further study. Third, validation were only conducted in a public database, and a prospective study with a substantial sample size is essential to confirm the clinical utility of the risk score.

## Conclusions

In short, we built a four-gene risk score model correlated with CD8 + T cell infiltration, which may provide some guidance for future prognosis prediction and molecular targeted therapy of STAD.

### Supplementary Information

**Additional file 1: Supplementary Figure 1.** Sensitivity to drugs in high- and low-risk groups of risk score. A-B: in TCGA training set; C-D: in GSE84437 validation set.

## Data Availability

The datasets generated during and/or analysed during the current study are available from the corresponding author on reasonable request. The TCGA-STAD dataset (https://xenabrowser.net/datapages/?dataset=TCGA-STAD.htseq_counts.tsv&host=https%3 A%2 F%2Fgdc.xenahubs.net&removeHub=https%3 A%2 F%2Fxena.treehouse.gi.ucsc.edu%3A443) was downloaded from UCSC Xena (https://xenabrowser.net/datapages/); GSE26899 dataset, GSE29272 dataset and GSE84437 dataset were downloaded from the GEO database (https://www.ncbi.nlm.nih.gov/geo/).
