# Supplementary material for: Evaluation of vital genes correlated with CD8 + T cell infiltration as prognostic biomarkers in stomach adenocarcinoma
Source: BMC Gastroenterol. 2023 Nov 17;23:399. doi: 10.1186/s12876-023-03003-y (PMC10656896; doi:10.1186/s12876-023-03003-y)
Supplement: Supplementary file 1 — Additional file 1: Supplementary Figure 1. Sensitivity to drugs in high- and low-risk groups of risk score. A-B: in TCGA training set; C-D: in GSE84437 validation set. [file 12876_2023_3003_MOESM1_ESM.docx]

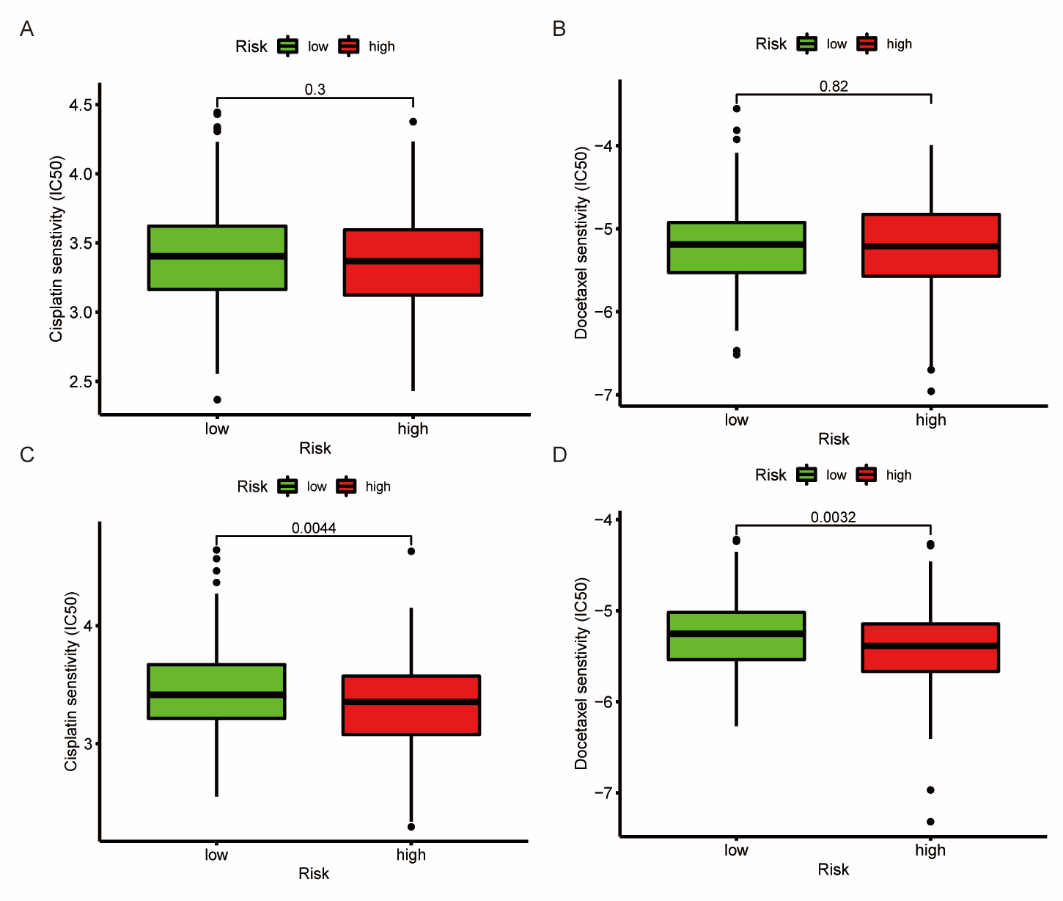


**Supplementary Figure 1:** Sensitivity to drugs in high- and low-risk groups of risk score.

A-B: in TCGA training set; C-D: in GSE84437 validation set.
